# A retrospective study on tuberculosis treatment outcomes at Jinka General Hospital, southern Ethiopia

**DOI:** 10.1186/s13104-017-3020-z

**Published:** 2017-12-04

**Authors:** Biniam Wondale, Girmay Medihn, Takele Teklu, Wondmeneh Mersha, Mesfin Tamirat, Gobena Ameni

**Affiliations:** 1 Aklilu Lemma Institute of Pathobiology, Addis Ababa University, Addis Ababa, Ethiopia; 2 Department of Biology, Arba Minch University, Arba Minch, Ethiopia; 3 Department of Immunology and Molecular Biology, University of Gonder, Gonder, Ethiopia; 4 Jinka General Hospital, Jinka, Ethiopia

**Keywords:** Tuberculosis, Treatment outcomes, DOTS, Jinka, South Omo

## Abstract

**Objective:**

Information on tuberculosis (TB) treatment outcomes would be useful for the improvement of the TB control program. The aim of the present study was to evaluate treatment outcomes of TB and identify associated factors in TB patients at the Jinka General Hospital (JGH), remote Zone of Ethiopia.

**Result:**

The result showed that 13.1% (154/1172) of the cases were cured, 60.9% treatment completed, 10.2% died and 9.1% were lost to follow-up. Thus, the overall treatment success rate was 74%. Male patients [AOR = *0.70* (0.52–0.93)] and HIV co-infected patients [AOR = *0.67* (0.45–0.98)] were associated with unsuccessful treatment outcomes.

**Electronic supplementary material:**

The online version of this article (10.1186/s13104-017-3020-z) contains supplementary material, which is available to authorized users.

## Introduction

Tuberculosis (TB) remains one of the health problems in the developing world even after the development of effective treatment [1]. TB becomes the leading cause of death from a single infectious disease [2]. Globally, 10.4 million new TB cases were recorded in 2015 [2], Africa accounting for 26% of the global TB [2].

Out of the 30 high TB burden countries, Ethiopia ranks 11th [2] and the prevalence of TB per 100,000 populations in Ethiopia was 482 in 1994 [3] and declined progressively to 200 in 2014 [4]. The estimate of TB incidence per 100,000 populations was 431 in 1997 which was declined to 192 in 2015 [2]. The prevalence of multidrug-resistant (MDR)-TB in Ethiopia has been increasing from 1.6 to 2.3% in new cases and 11.8–17.8% in previously treated TB cases from 2005 to 2014 [5]. In addition, 17.6% of all TB patients in Ethiopia were co-infected with HIV [5].

The Federal Ministry of Health of Ethiopia (FMOH) has been implementing a number of strategies specific to TB, TB/HIV, and MDR-TB. Direct Observed Treatment Short-course (DOTS) was recommended by WHO since the mid-1990s. DOTS has been started in Ethiopia in 1992 as a pilot [6, 7] and since then Ethiopia has been implementing DOTS.

So far, TB treatment outcomes in Ethiopia were evaluated in a limited number of easily accessible health facilities [8–25]. However, treatment outcomes are dependent on adherence to the treatment protocol which is also dependent on the knowledge and commitment of patients and health professionals [26]. This implies that patients who live in less accessible and geographically distant places could have low level of knowledge that may contribute to the reduced adherence to treatment [27, 28].

Studies indicated that treatment success rate (TSR) in Ethiopia varies from 26% [11] to 94.4% [13] depending on different factors. Therefore, evaluating the treatment outcomes of specific localities such as that of the South Omo Zone (SOZ) could play an important role in addressing the TB control problems of the Zone. The objective of this study was, therefore, to evaluate the TB treatment outcomes and identify associated risk factors in the Jinka General Hospital (JGH), southern Ethiopia.

## Main text

### Materials and methods

#### Study settings

The study was carried out using data extracted from medical records of TB patients registered in the JGH between July 2004 and June 2014. The JGH is located in SOZ, southern Ethiopia. According to the 2007 census, the total population of the SOZ was 577,673 (7.5% urban and 92.5% rural) of whom 50% were men [29]. In the SOZ, the first health center was established in April 1962 (Hospital archive). Presently, the JGH, the only Hospital in the SOZ, has been serving the community since April 2001.

Direct Observed Treatment Short-course was introduced in the Southern Nations and Nationalities People Region (SNNPR), in 1996 [30] and started in the SOZ in 2001 at the JGH setting (personal communication). Its effectiveness has never been evaluated. The JGH uses first-line TB drugs in a combination of two or more drugs which includes streptomycin (S), ethambutol (E), isoniazid (H), rifampicin (R) and pyrazinamide (Z). Treatment regimens were: short course chemotherapy (SCC)—2(RHZ) plus or minus E or S/followed by 6(EH) or 4(RH) for new patients; 2S(RHZE), 1(RHZE) and 5(RHE) for re-treatment patients, and Long course chemotherapy (LCC)—2S(HE)/10(RE) for patients with serious liver disease [31]. Since 2009, the continuation phase regimen has been changed from 6(EH) to 4(RH) [32].

#### Definitions

Tuberculosis patients clinically and, before and after TB treatment were categorized based on guidelines of Ethiopian TB, TB/HIV and leprosy control program and WHO [33, 34].

#### Data collection

Tuberculosis treatment registers in JGH from July 2004 to June 2014 were used as the data source. The data collected included year of treatment, sex, age, treatment history, type of TB, HIV test result and treatment outcomes. The collected data were entered into an Excel spreadsheet and the data were cross-checked for the correctness before analysis.

#### Data analysis

Data analysis was made by transforming the data from Excel into IBM SPSS Statistics 20. The results were presented using descriptive statistics. The associations between TB treatment outcomes and independent variables were computed using binary logistic regression. Crude odds ratio (COR) and adjusted odds ratio (AOR) were used to present the results. value less than 0.05 was considered statistically significant.

### Results

#### Demographic and clinical characteristics of TB patients

Demographic and clinical characteristics of TB patients were summarized in Table 1. A total of 2156 TB patients (59.4% male) with the age range of 0.25–95 years were registered during the 10 years. The mean, standard deviation and median age of the TB patients were 30.1, 15.4 and 29, respectively. The highest percentage (77.7%) of the cases was in the age range 15–54 years. The proportion of smear-negative pulmonary TB (PTB−) cases, new cases, re-treatment cases and transfer-in cases were 48.4, 92.3, 5.3 and 2.3% respectively. TB/HIV co-infection was 19.6% (315/1604) and higher percentage was recorded in smear-negative PTB (PTB−) patients.

**Table 1:** Demographic characteristics of TB patients registered at JGH (July 2004–June 2014)

Characteristics	Number (%) stratified by type of TB			Total N (%)
	PTB−	PTB+	EPTB	
Sex				
Female	420 (40.2)	225 (40.7)	231 (41.3)	876 (40.6)
Male	624 (59.8)	328 (59.3)	328 (58.7)	1280 (59.4)
Age in years				
< 15	150 (14.4)	31 (5.6)	119 (21.3)	300 (13.9)
15–24	137 (13.1)	149 (26.9)	132 (23.6)	418 (19.4)
25–34	297 (28.4)	202 (36.5)	163 (29.2)	662 (30.7)
35–44	219 (21.0)	88 (15.9)	80 (14.3)	387 (17.9)
45–54	116 (11.1)	60 (10.8)	32 (5.7)	208 (9.6)
≥ 55	125 (12.0)	23 (4.2)	33 (5.9)	181 (8.4)
TB patients category				
New TB cases	976 (93.5)	495 (89.5)	520 (93.0)	1991 (92.3)
Re-treatment TB cases	53 (5.1)	37 (6.7)	25 (4.5)	115 (5.3)
Transfer-in TB cases	15 (1.4)	21 (3.8)	14 (2.5)	50 (2.3)
HIV test result				
Non reactive	602 (57.7)	331 (59.9)	356 (63.7)	1289 (59.8)
Reactive	209 (20.0)	69 (12.5)	37 (6.6)	315 (14.6)
Unknown	233 (22.3)	153 (27.7)	166 (29.7)	552 (25.6)
Year of registration for TB treatment				
July 2004–June 2005	25 (2.4)	48 (8.7)	30 (5.4)	103 (4.8)
July 2005–June 2006	57 (5.5)	27 (4.9)	53 (9.5)	137 (6.4)
July 2006–June 2007	82 (7.9)	39 (7.1)	56 (10.0)	177 (8.2)
July 2007–June 2008	104 (10.0)	47 (8.5)	50 (8.9)	201 (9.3)
July 2008–June 2009	102 (9.8)	46 (8.3)	31 (5.5)	179 (8.3)
July 2009–June 2010	124 (11.9)	43 (7.8)	31 (5.5)	198 (9.2)
July 2010–June 2011	189 (18.1)	41 (7.4)	70 (12.5)	300 (13.9)
July 2011–June 2012	224 (21.5)	92 (16.6)	87 (15.6)	403 (18.7)
July 2012–June 2013	61 (5.8)	71 (12.8)	72 (12.9)	204 (9.5)
July 2013–June 2014	76 (7.3)	99 (17.9)	79 (14.1)	254 (11.8)
Total	1044 (48.4)	553 (25.6)	559 (25.9)	2156 (100)

#### Treatment outcomes

Treatment outcomes of the cases are summarized in Table 2. Out of 2156 TB patients registered at the JGH between 2004 and 2014, 45.6% (984/2156) was transferred out. Treatment outcomes were available only for 54.4% (1172/2156) of the patients. Out of the later, 13.1, 60.9, 10.2 and 9.1% were cured, treatment completed, died and loss to follow-up, respectively. TSR was 91.5% (43/47), 80.8% (227/281), 78.9% (422/535) and 84.1% (53/63) in transfer-in, smear-positive pulmonary TB (PTB+), HIV negative, and July 2012–June 2013 registered cases respectively. The cure rates were 54.8% (154/281) and 67.8% (154/227) in all cases and PTB+ cases, respectively. Higher numbers of unsuccessful treatment outcomes were recorded in PTB− cases. The TSR showed an increasing trend in the first 5 years and then a declining trend except during 1 year (July 2012 and June 2013).

**Table 2:** Treatment outcomes of TB patients in JGH (July 2004–June 2014)

Characteristics	Number (%) stratified by the treatment outcomes						Total N (%)	P value
	Successful treatment outcomes 868 (74)		Unsuccessful treatment outcomes 304 (24)					
	Cured	Treatment completed	Died	T. failure^a^	Loss to follow-up	Interrupter		
Sex								
Female	66 (14.2)	299 (64.3)	40 (8.6)	0 (0.0)	30 (6.5)	30 (6.5)	465 (39.7)	0.059
Male	88 (12.4)	415 (58.7)	79 (11.2)	1 (0.1)	77 (10.9)	47 (6.6)	707 (60.3)	
Age in years								
< 15	5 (3.4)	101 (69.7)	10 (6.9)	0 (0.0)	8 (5.5)	21 (14.5)	145 (12.4)	< 0.001
15–24	55 (21.7)	145 (57.1)	19 (7.5)	0 (0.0)	22 (8.7)	13 (5.1)	254 (21.7)	
25–34	56 (15.3)	216 (59.2)	39 (10.7)	1 (0.3)	34 (9.3)	19 (5.2)	365 (31.1)	
35–44	23 (10.6)	138 (63.3)	24 (11.0)	0 (0.0)	22 (10.1)	11 (5.0)	218 (18.6)	
45–54	10 (10.4)	50 (52.1)	16 (16.7)	0 (0.0)	12 (12.5)	8 (8.3)	96 (8.2)	
≥ 55	5 (5.3)	64 (68.1)	11 (11.7)	0 (0.0)	9 (9.6)	5 (5.3)	94 (8.0)	
TB patients category								
New TB cases	125 (12.0)	633 (61.0)	107 (10.3)	0 (0.0)	98 (9.4)	75 (7.2)	1038 (88.6)	< 0.001
Re-treatment TB cases	16 (30.2)	22 (51.2)	5 (11.6)	0 (0.0)	3 (7.0)	0 (0.0)	43 (3.7)	
Transfer-in TB cases	13 (27.7)	30 (63.8)	1 (2.1)	0 (0.0)	2 (4.3)	1 (2.1)	47 (4.0)	
Type of TB								
PTB−	NA	436 (71.5)	72 (11.8)	NA	51 (8.4)	51 (8.4)	610 (52.0)	< 0.001
PTB+	154 (54.8)	73 (26.0)	24 (8.5)	1 (0.4)	22 (7.8)	7 (2.5)	281 (24.0)	
EPTB	NA	205 (73.0)	23 (8.2)	NA	34 (12.1)	19 (6.8)	281 (24.0)	
HIV test result								
Non reactive	81 (15.1)	341 (63.7)	29 (5.4)	0 (0.0)	39 (7.3)	45 (8.4)	535 (45.6)	< 0.001
Reactive	28 (11.9)	138 (58.7)	41 (17.4)	0 (0.0)	15 (6.4)	13 (5ρ.5)	235 (20.1)	
Unknown	45 (11.2)	235 (58.5)	49 (12.2)	1 (0.2)	53 (13.2)	19 (4.7)	402 (34.3)	
Year of TB treatment								
July 2004–June 2005	10 (12.2)	43 (52.4)	14 (17.1)	1 (1.2)	13 (15.9)	1 (1.2)	82 (7.0)	< 0.001
July 2005–June 2006	9 (8.3)	63 (57.8)	21 (19.3)	0 (0.0)	15 (13.8)	1 (0.9)	109 (9.3)	
July 2006–June 2007	13 (8.9)	91 (62.3)	11 (7.5)	0 (0.0)	21 (14.40)	10 (6.8)	146 (12.5)	
July 2007–June 2008	18 (11.8)	101 (66.0)	19 (12.4)	0 (0.0)	10 (6.5)	5 (3.3)	153 (13.1)	
July 2008–June 2009	20 (16.3)	83 (67.5)	11 (8.9)	0 (0.0)	4 (3.3)	5 (4.1)	123 (10.5)	
July 2009–June 2010	19 (13.7)	92 (66.2)	12 (8.6)	0 (0.0)	12 (8.6)	4 (2.9)	139 (11.9)	
July 2010–June 2011	21 (11.7)	120 (67.0)	8 (4.5)	0 (0.0)	8 (4.5)	22 (12.3)	179 (15.3)	
July 2011–June 2012	15 (14.3)	57 (54.3)	6 (5.7)	0 (0.0)	8 (7.6)	19 (18.1)	105 (9.0)	
July 2012–June 2013	19 (30.2)	34 (54.0)	6 (9.5)	0 (0.0)	1 (1.6)	3 (4.8)	63 (5.4)	
July 2013–June 2014	10 (13.7)	30 (41.1)	11 (15.1)	0 (0.0)	15 (20.5)	7 (9.6)	73 (6.2)	
Total	154 (13.1)	714 (60.9)	119 (10.2)	1 (0.1)	107 (9.1)	77 (6.6)	1172 (100)	

*NA* not applicable, *T. failure* ^a^ treatment failure

#### Treatment success and associated factors of TB patients

Multiple logistic regression analysis indicated that male (AOR = 0.70, 95% CI 0.52–0.93) and TB/HIV co-infection (AOR = 0.67, 95% CI 0.46–0.98) were significantly associated with reduced odds of treatment success (Table 3). While patients who transferred-in (AOR = 3.80, 95% CI 1.32–10.94) and PTB+ (AOR = 1.81, 95% CI 1.24–2.64) were significantly associated with the odds of having better treatment success.

**Table 3:** Factors associated with treatment success rate TB cases at JGH (July 2004–June 2014)

Characteristics	Total N (%)	Treatment success N (%)	Crude OR (95% CI)	Adjusted OR (95% CI)	P value
Sex					
Female	465 (39.7)	365 (78.5)	1.00	1.00	
Male	707 (60.3)	503 (71.1)	*0.68 (0.51–0.89)*	*0.70 (0.52–0.93)*	*0.013*
Age in years					
< 15	145 (12.4)	106 (73.1)	1.00	1.00	
15–24	254 (21.7)	200 (78.7)	1.36 (0.85–2.19)	1.30 (0.79–2.14)	0.301
25–34	365 (31.1)	272 (74.5)	1.08 (0.70–1.66)	1.18 (0.74–1.87)	0.493
35–44	218 (18.6)	161 (73.9)	1.04 (0.65–1.67)	1.13 (0.68–1.86)	0.645
45–54	96 (8.2)	60 (62.5)	0.61 (0.35–1.07)	0.63 (0.35–1.13)	0.122
≥ 55	94 (8.0)	69 (73.4)	1.02 (0.57–1.83)	1.04 (0.56–1.91)	0.912
TB patients category					
New TB cases	1038 (88.6)	758 (73.0)	1.00	1.00	
Re-treatment TB cases	87 (7.4)	67 (77.0)	1.24 (0.74–2.08)	0.94 (0.55–1.62)	0.831
Transfer-in TB cases	47 (4.0)	43 (91.5)	*3.97 (1.41–11.16)*	*3.79 (1.31–10.92)*	*0.014*
Type of TB					
PTB−	610 (52.0)	436 (71.5)	1.00	1.00	
PTB+	281 (24.0)	227 (80.8)	*1.68 (1.19–2.37)*	*1.81 (1.24–2.64)*	*0.002*
EPTB	281 (24.0)	205 (73.0)	1.08 (0.78–1.48)	1.19 (0.84–1.68)	0.328
HIV test result					
Non reactive	535 (45.6)	422 (78.9)	1.00	1.00	
Reactive	235 (20.1)	166 (70.6)	*0.64 (0.45–0.91)*	*0.67 (0.45–0.98)*	*0.036*
Unknown	402 (34.3)	280 (69.7)	*0.62 (0.46–0.83)*	0.81 (0.44–1.49)	0.492
Year of registration for TB treatment					
July 2004–June 2005	82 (7.0)	53 (64.6)	1.00	1.00	
July 2005–June 2006	109 (9.3)	72 (66.1)	1.07 (0.58–1.94)	1.17 (0.63–2.18)	0.614
July 2006–June 2007	146 (12.5)	104 (71.2)	1.36 (0.76–2.41)	1.62 (0.89–2.93)	0.114
July 2007–June 2008	153 (13.1)	119 (77.8)	*1.92 (1.06–3.46)*	*2.29 (1.03–5.09)*	*0.043*
July 2008–June 2009	123 (10.5)	103 (83.7)	*2.82 (1.46–5.45)*	*3.29 (1.38–7.84)*	*0.007*
July 2009–June 2010	139 (11.9)	111 (79.9)	*2.17 (1.17–4.01)*	*2.53 (1.11–5.75)*	*0.027*
July 2010–June 2011	179 (15.3)	141 (78.8)	*2.03 (1.14–3.62)*	*2.37 (1.03–5.45)*	*0.042*
July 2011–June 2012	105 (9.0)	72 (68.6)	1.19 (0.65–2.20)	1.36 (0.59–3.14)	0.474
July 2012–June 2013	63 (5.4)	53 (84.1)	*2.90 (1.29–6.54)*	*2.78 (1.01–7.66)*	*0.048*
July 2013–June 2014	73 (6.2)	40 (54.8)	0.66 (0.35–1.27)	0.65 (0.26–1.60)	0.348

Italic values to show significance

### Discussion

In the present study, the records of TB cases registered at JGH between 2004 and 2014 were extracted and analyzed for the evaluation of the treatment outcomes. The analysis was done for 54.4% (1172/2156) of TB cases. The ratio of male to female cases was 1.5:1, which is in agreement with the reports of WHO [2] and with those of the previous studies in Ethiopia [11, 14, 16, 23, 24] and in other countries [35–39]. The larger number of TB cases in males than females could be due to biological differences, the difference in societal roles and access to health facilities [40, 41].

The finding of this study showed that the percentage of TB was highest in the productive age group (15–54) and it was in agreement with the results of studies conducted in Ethiopia [8, 14, 25] and Bhutan [42]. Such findings could be due to the greater mobility of this age group for economic and social reasons.

TB/HIV co-infection recorded in the present study was higher than the average percentages of Ethiopia and that of the Southern region of Ethiopia [5]. Moreover, it was higher than those of other studies in Ethiopia [9, 10, 13, 18, 23] and in Spain [37]. On the other hand, other studies [8, 11] reported higher percentages of TB/HIV co-infection compared to that of the present study. As suggested by an earlier study, the variation in the percentage of TB/HIV co-infection is mainly attributed to the prevalence of HIV in the study population [43]. In this study, PTB− cases had higher number of TB/HIV co-infection than PTB+ cases. This is the fact that TB cases with HIV are less likely to be smear-positive [44].

The TSR recorded, 74%, by this study was less than the target to be achieved, 87%, by 2015 [45]. It was also less than the TSR reported from the other regions of Ethiopia [9, 10, 13, 15, 21, 24]. On the other hand, it was similar with that of one study [11] and greater than that of reporting from the northern Ethiopia [22] and greater than that reported from Nigeria [39]. Such variations in TSR could be attributed to differences in socio-economic of the patients, geographic setting, sample size, study period and the TB clinic management.

In the present study, 32.2% of PTB+ cases among who adhere and complete their treatment did not provide sputum for AFB examination according to the guideline [33] or their smear results were not recorded. This could be due to lack of commitment of health professionals and/or poor awareness of PTB+ patients for checking their status during treatment.

During the study period, there was an increment of TSR for the first 5 years and then decline between July 2012 and June 2013. The decline of TSR could be due to lack of continues support/encouragement for people working in TB clinic and/or poor awareness of TB patients.

Male and TB/HIV co-infected cases demonstrated lower treatment success and this was in agreement with the findings reported in Ethiopia [10, 16, 19, 22] and in Malawi [46]. Low treatment success in males could be attributed to risk-taking behavior like use of tobacco, alcohol and illicit drug [47] and in TB/HIV co-infected patients could be due to co-administration of ART along with anti-TB therapy which can lead to drug–drug interactions, overlapping drug toxicities and immune reconstitution syndrome [48].

In agreement with the studies in Ethiopia [22] and in Malawi [49], PTB+ cases in this study was associated with a higher treatment success which could be due to the easier accessibility of the drugs to the TB bacilli as the granuloma is usually burst in PTB+ cases and could also be due to more number of TB/HIV co-infected cases and higher number of death in PTB− patients. In addition, transfer-in patients had high probability of treatment success. It could be due to patients desire to be in an ideal health facility to continue and complete their treatment.

The percentage of overall unsuccessful treatment outcomes reported by the present study was similar with some studies [12, 17, 22] while greater than other studies [8–11, 13–16, 18, 20, 21, 23, 24]. Greater numbers of unsuccessful TB cases in the present study could be attributed to being in a remote area which leads to poor knowledge and having poor socio-economic status together reduces treatment adherence [27, 28].

### Conclusion and recommendations

The TSR recorded in the present study was less than the target to be met by 2015. This implies the necessity of urgent management response to improve current TSR. The low percentage of cure rate in PTB+ patients could suggest the strengthening of services of TB Clinic of the JGH. This is an area that needs health system level intervention. HIV positivity and male gender were associated with poor treatment success which requires targeted TB control management. Hence, improvement and strengthening of comprehensive and targeted TB–HIV control program are recommended to the study area.

## Limitations

This study had the following limitations and born from being secondary data: lack of HIV status for the significant number of cases that could affect treatment outcomes, the low percentage of PTB+ cases which could be attributed to negligence or poor skills of the technicians, inadequate sputum examination records for PTB+ cases during treatment which could be due to the negligence of health professionals and could be a reason for low cure rate and for only one treatment failure case, and finally a high percentage of transfer out cases which could be attributed to the long distance between the Hospital and living districts of the patients which obliges the transfer out of the patients so that they can be treated at the nearby health center.

## Additional file

**Additional file 1.** 10 years (2004–2014) TB DOTS data of JGH, Ethiopia. This is a file which contains information has been used for data analysis.

## Abbreviations

AOR: adjusted odds ratio
COR: crude odds ratio
DOTS: Direct Observed Treatment Short-course
JGH: Jinka General Hospital
MDR: multi-drug resistant
PTB−: smear-negative pulmonary TB
PTB+: smear-positive pulmonary TB
SOZ: South Omo Zone
TB: tuberculosis

## References

[1] Sia IG, Wieland ML (2011). Current concepts in the management of tuberculosis. Mayo Clin Proc.

[2] World Health Organization (2016). Global tuberculosis report 2016.

[3] Federal Democratic Republic of Ethiopia Minister of Health. 16th National Annual Review Meeting Group Discussion: why TB? Evaluating the National TB Control Program: challenges and ways forward. ARM 16-Doc 07/14. October 2014. Addis Ababa, Ethiopia; 2014.

[4] World Health Organization (2015). Global tuberculosis report 2015.

[5] Ethiopian Public Health Institute and Federal Democratic Republic of Ethiopia Minster of Health. National TB/HIV sentinel surveillance annual report (July 2014–June 2015) December 2015. Addis Ababa, Ethiopia; 2015.

[6] World Health Organization (1999). What is DOTS? A guide to understanding the WHO-recommended TB control strategy known as DOTS 1999.

[7] Federal Democratic Republic of Ethiopia Minster of Health and Ethiopian Public Health Institute. Impact Evaluation of Ethiopia’s National Response to HIV/AIDS, Tuberculosis and Malaria 2008. Addis Ababa, Ethiopia; 2008.

[8] Addis Z, Birhan W, Alemu A, Mulu A, Ayal G, Negash H (2013). Treatment outcome of tuberculosis patients in Azezo Health Center, North West Ethiopia. IJBAR.

[9] Berhe G, Enquselasse F, Aseffa A (2012). Treatment outcomes of smear-positive pulmonary tuberculosis patients in Tigray Region, northern Ethiopia. BMC Public Health.

[10] Beza MG, Wubie MT, Teferi MD, Getahun YS, Bogale SM, Tefera SB (2013). A five years tuberculosis treatment outcome at Kolla Diba Health Center, Dembia District, Northwest Ethiopia: a retrospective cross-sectional analysis. J Infect Dis Ther.

[11] Biadglegne F, Anagaw B, Debebe T, Anagaw B, Tesfaye W, Tessema B, Rodloff AC, Sack U (2013). A retrospective study on the outcomes of tuberculosis treatment in Felege Hiwot Referal Hospital, Nothwest Ethiopia. Int J Med Med Sci.

[12] Ejeta E, Birhanu T, Wolde T (2014). Tuberculosis treatment outcomes among tuberculosis/human immunodeficiency co-infected cases treated in under direct observed treatment of short course in western Ethiopia. J AIDS HIV Res.

[13] Gebreegziabher SB, Yimer SA, Bjune GA (2016). Tuberculosis case notification and treatment outcomes in west Gojjam Zone, Northwest Ethiopia: a five-year retrospective study. J Tuberc Res.

[14] Gebrezgabiher G, Romha G, Ejeta E, Asebe G, Zemene E, Ameni G (2016). Treatment outcome of tuberculosis patients under direct observed treatment short course and factors affecting outcome in southern Ethiopia: a five year retrospective study. PLoS ONE.

[15] Getahun B, Ameni G, Medhin G, Biadgilign S (2013). Treatment outcome of tuberculosis patients under directly observed treatment in Addis Ababa, Ethiopia. Braz J Infect Dis.

[16] Jemal M, Tarekegne D, Atanaw T, Ebabu A, Endris M, Tessema B, Moges F, Deressa T (2015). Treatment outcomes of tuberculosis patients in Metema Hospital, Northwest Ethiopia: a four years retrospective study. Mycobact Dis.

[17] Lindtjorn B, Madebo T (2001). The outcome of tuberculosis treatment at a rural hospital in southern Ethiopia. Trop Dr.

[18] Ramos JM, Reyes F, Tesfamariam A (2010). Childhood and adult tuberculosis in a rural hospital in Southeast Ethiopia: a ten-year retrospective study. BMC Public Health.

[19] Shargie EB, Lindtjorn B (2005). DOTS improves treatment outcomes and service coverage for tuberculosis in South Ethiopia: a retrospective trend analysis. BMC Public Health.

[20] Sisay S, Mengistu B, Erku W, Woldeyohannes D (2014). Direct observed treatment short-course (DOTS) for tuberculosis control program in Gambella Regional State, Ethiopia: ten years experience. BMC Res Notes.

[21] Tadesse S, Tadesse T (2014). Treatment success rate of tuberculosis patients in Dabat, northwest Ethiopia. Health.

[22] Tessema B, Muche A, Bekele A, Reissig D, Emmrich F, Sack U (2009). Treatment outcome of tuberculosis patients at Gonder University Teaching Hospital, Northwest Ethiopia. A five-year retrospective study. BMC Public Health.

[23] Sintayehu W, Abera A, Gebru T, Fiseha T (2014). Trends of tuberculosis treatment outcomes at Mizan-Aman general hospital, southwest Ethiopia: a retrospective study. Int J Immunol.

[24] Woldeyohannes D, Sisay S, Mengistu B, Kassa H (2015). Direct observed treatment short-course (DOTS) for treatment of new tuberculosis cases in Somali Regional State, eastern Ethiopia: ten years retropspective study. BMC Res Notes.

[25] Yassin MA, Datiko DG, Tulloch O, Markos P, Aschalew M, Shargie EB, Dangisso MH, Komatsu R, Sahu S, Blok L, Cuevas LE, Theobald S (2013). Innovative community based approaches doubled tuberculosis case notification and improve treatment outcome in southern Ethiopia. PLoS ONE.

[26] Martin LR, Williams SL, Haskard KB, DiMatteo MR (2005). The challenge of patient adherence. Ther Clin Risk Manag.

[27] Tadesse T, Demissie M, Berhane Y, Kebede Y, Abebe M (2013). Long distance travelling and financial burdens discourage tuberculosis DOTS treatment initiation and compliance in Ethiopia: a qualitative study. BMC Public Health.

[28] Nezenega ZS, Gacho YH, Tafere TE (2013). Patient satisfaction on tuberculosis treatment service and adherence to treatment in public health facilities of Sidama zone, South Ethiopia. BMC Health Serv Res.

[29] Central Statistic Agency of Ethiopia. Summary and statistical report of the 2007 population and housing census-population size by age and sex, Federal Democratic Republic of Ethiopia, Population Census Commission, with support from UNFPA. Addis Ababa, Ethiopia; 2008.

[30] Yassin MA, Takele L, Gebresenbet S, Girma E, Lera M, Lendebo E, Cuevas LE (2004). HIV and tuberculosis co-infection in the southern region of Ethiopia: a prospective epidemiological study. Scand J Infect Dis.

[31] Drug Administration and Control Authority of Ethiopia. Standard treatment guidelines for district hospital—Ethiopia. Addis Ababa, Ethiopia; 2004.

[32] Federal Minister of Health Ethiopia. Interim guideline for shifting from EH to RH Regimen. Addis Ababa, Ethiopia; 2010.

[33] Federal Democratic Republic of Ethiopia Minster of Health. Guidelines for clinical and programmatic management of TB, TB/HIV and Leprosy in Ethiopia. Addis Ababa, Ethiopia; 2013.

[34] World Health Organization (2013). Definitions and reporting framework for tuberculosis—2013 revision.

[35] Anunnatsiri S, Chetchotisakd P, Wanke C (2005). Factors associated with treatment outcomes in pulmonary tuberculosis in Northeastern Thailand. Southeast Asia J Trop Med Public Health.

[36] Van den Boogaard J, Lyimo R, Irongo CF, Boeree MJ, Schaalma H, Aarnoutse RE, Kibiki GS (2009). Community vs. facility-based directly observed treatment for tuberculosis in Tanzania’s Kilimanjaro Region. Int J Tuberc Lung Dis.

[37] Cayla JA, Caminero JA, Rey R, Lara N, Valles X, Galdos-Tanguis H (2004). Current status of treatment completion and fatality among tuberculosis patients in Spain. Int J Tuberc Lung Dis.

[38] Jibrin YB, Ali AB, Saad ST, Kolo PM (2013). Prevalence of treatment failure among pulmonary tuberculosis patients in Federal Medical Centre, Gombe, Northeastern Nigeria. ISRN Infect Dis.

[39] Babatunde OA, Elegbede OE, Ayodele M, Fadare JO, Isinjaye AO, Ibirongbe DO, Akinyandenu J (2013). Factors affecting treatment outcomes of tuberculosis in a tertiary health center in southwestern Nigeria. Int Rev Soc Sci Humanit.

[40] World Health Organization (2009). Global tuberculosis control: epidemiology, strategy, financing 2009.

[41] Borgdorff MW, Nagelkerke NJD, Dye C, Nunn P (2000). Gender and tuberculosis: a comparison of prevalence surveys with notification data to explore sex differences in case detection. Int J Tuberc Lung Dis.

[42] Wangdi K, Gurung MR (2012). The epidemiology of tuberculosis in Phuentsholing General Hospital: a six year retrospective study. BMC Res Notes.

[43] Datiko DG, Yassin MA, Chekol LT, Kabeto LE, Lindtjorn B (2008). The rate of TB-HIV co-infection depends on the prevalence of HIV infection in the community. BMC Public Health.

[44] Corbett EL, Watt CJ, Walker N, Maher D, Williams BG, Raviglione MC, Dye C (2003). The growing burden of tuberculosis: global trend and interactions with the HIV epidemic. Arch Intern Med.

[45] Stop TB Partnership Secretariat. The global plan to stop TB 2011–2015: transforming the fight towards elimination of tuberculosis.

[46] Tweya H, Feldacker C, Phiri S, Ben-Smith A, Fenner L, Jahn A, Kalulu M, Weigel R, Kamba C, Banda R, Egger M, Keiser O (2013). Comparison of treatment outcomes of new smear-positive pulmonary tuberculosis patients by HIV and antiretroviral status in a TB/HIV Clinic, Malawi. PLoS ONE.

[47] Thom E (2003). Risk-taking behaviour in men: Substance use and gender.

[48] Padmapriyadarsini C, Narendran G, Swaminathan S (2011). Diagnosis and treatment of tuberculosis in HIV co-infected patients. Indian J Med Res.

[49] Harries AD, Nyirenda TE, Banerjee A, Boeree MJ, Salaniponi FML (1999). Treatment outcome of patients with smear negative and smear positive pulmonary tuberculosis in the national tuberculosis programme, Malawi. Trans R Soc Trop Med Hyg.

